# Adrenal hemorrhage and myocarditis in a patient with systemic lupus erythematosus: A case report and literature review

**DOI:** 10.1016/j.radcr.2025.06.023

**Published:** 2025-06-26

**Authors:** Swara H. Abdullah, Dilan S. Hiwa, Lawen Jamal Mustafa, Farman J. Ahmed, Shaho F. Ahmed, Dana H. Mohammed-Saeed, Aso N. Qadir, Jamal M. Salih, Hemin S. Mohammed, Fahmi H. Kakamad

**Affiliations:** a Department of Medicine, Shar Hospital, Sulaymaniyah, Kurdistan, Iraq; b Scientific Affairs Department, Smart Health Tower, Sulaymaniyah, Kurdistan, Iraq; c Rheumatology Department, Ministry of Health, Sulaymaniyah, Kurdistan, Iraq; d College of Medicine, University of Sulaimani, Sulaymaniyah, Kurdistan, Iraq; e Scientific Department, Xzmat Polyclinic, Rizgari, Kalar, Sulaymaniyah, Kurdistan, Iraq; f Kscien Organization for Scientific Research (Middle East Office), Sulaymaniyah, Kurdistan, Iraq

**Keywords:** Adrenal insufficiency, Lupus myocarditis, SLE, Connective tissue disease, Diagnosis

## Abstract

Bilateral adrenal hemorrhage is a rare and serious complication that might affect patients with concomitant systemic lupus erythematosus (SLE) and antiphospholipid syndrome (APS). However, this study presents a rare case of SLE without APS that developed bilateral adrenal hemorrhage, causing adrenal insufficiency and later complicated by lupus myocarditis, resulting in rapid deterioration and death. A 35-year-old female complained of generalized body aches associated with arthralgia, joint swelling, abdominal pain, and fatigue for 2 weeks duration. The clinical course was complicated by seizure, bilateral adrenal hemorrhage, and resultant adrenal insufficiency in addition to pulmonary embolism. High troponin titers and echocardiographic findings of global wall hypokinesia suggested lupus myocarditis. Despite treatment with multiple medications throughout the patient's course of disease, including hydrocortisone, methylprednisolone, and hydroxychloroquine, the patient's condition deteriorated, and she died. Only a few cases of isolated SLE complicated by adrenal hemorrhage were reported during the literature review, with the majority of other cases associated with APS. Notably, no instances of concurrent myocarditis were reported in the reviewed studies. Adrenal hemorrhage may result from heightened arterial or venous pressure, often linked to elevated adrenocorticotropic hormone levels during stress or hypercoagulability. Anticoagulants can increase this risk, while reperfusion injury after hypotensive episodes also contributes. Patients with an SLE flare can develop bilateral adrenal hemorrhage and cause adrenal insufficiency even in the absence of definitive APS. The development of lupus myocarditis in such patients may result in worsened outcomes and potentially death.

## Background

Systemic lupus erythematosus (SLE) is a chronic autoimmune condition characterized by cycles of flare-ups and remissions, presenting a diverse range of symptoms that can result in a spectrum of severity from mild to severe [1]. A significant gender disparity is exhibited in SLE patients, with approximately ten females diagnosed for every male affected by the condition, with the majority of individuals recorded being middle-aged females in community-based Caucasian registries. Global adjusted prevalence rates are reaching, and in some instances surpassing, the range of 50-100 per 100,000 adults [2]. The primary symptoms reported by most SLE patients in the early stages are related to the skin, mucous membranes, and musculoskeletal system. Nevertheless, any system such as the renal, respiratory, cardiovascular, neuropsychiatric and hematological systems may be involved [1]. One of the cardiovascular manifestations is lupus myocarditis, with its clinical manifestation being an infrequent occurrence that may display signs of heart failure, including dyspnea, resting tachycardia, fever, chest discomfort, and/or myopericarditis [3]. Symptoms of SLE may not all appear concurrently, with months or even years passing between the onset of different manifestations [1]. In patients with SLE, several conditions are associated with it, most notably antiphospholipid syndrome (APS), which is a systemic autoimmune disorder distinguished by arterial, venous, or microvascular thrombotic events, obstetric complications during pregnancy in individuals demonstrating persistent antiphospholipid antibodies [4]. Approximately 20%-30% of individuals diagnosed with SLE exhibit antiphospholipid antibodies in their serum [5].

Adrenal hemorrhage, first documented in 1863 by Canton, is a rare but potentially deadly occurrence that can present in nontraumatic and traumatic contexts. The clinical manifestations of adrenal hemorrhage can exhibit significant variability, contingent upon factors such as the extent and pace of the hemorrhage, along with the degree of the adrenal cortex affected by the hemorrhagic event. In the uncommon occurrence of bilateral adrenal hemorrhage, adrenal insufficiency can arise when 90% of the adrenal cortex is compromised. Undiagnosed or delayed diagnosis of these instances can lead to death [6].

Although several case reports address bilateral adrenal hemorrhage in patients with concomitant SLE and APS, only a few cases of isolated SLE have been reported. In addition, there are no universally established treatment guidelines regarding this condition. We herein present a case of bilateral adrenal hemorrhage causing adrenal insufficiency in an SLE patient with concurrent lupus myocarditis, resulting in deterioration and death of the patient. The Kscien’s list was employed as a tool to discern and exclude publications from predatory journals while conducting the literature review and the report was written in accordance with CaReL guidelines [7,8].

## Case presentation

### Patient information

A 35-year-old female presented to the hospital on December 21, 2023 due to generalized body aches, weakness, and fatigue for 2 weeks duration. Her condition was associated with abdominal pain, anorexia, nausea, vomiting, and significant weight loss. The patient also complained of joint pain in multiple locations and swelling of hands. The joint pain initially began in her back and then progressed to the shoulders, elbows, wrists, metacarpophalangeal, proximal interphalangeal, and distal Interphalangeal joints. Subsequently, she also developed knee pain and was unable to walk. Upon further questioning, she also complained of palpitations, mild lower chest pain, and a dry cough with shortness of breath.

The patient was a nonsmoker and did not drink alcohol, but she had a significant past medical history of 2 miscarriages (one of them before ten weeks and the other one after 16 weeks of gestation) and migraine. Her surgical history was significant for rhinoplasty, appendectomy, and tonsillectomy in addition to 3 cesarian section deliveries in 2012, 2018, and the last in November of 2023. She was prescribed aspirin and folate for her pregnancy, but she stopped taking them 2-3 months prior to delivery. Her family’s medical history was insignificant.

### Clinical findings

The patient was alert but looked tired, with generalized dark skin compared to before her last pregnancy and pale conjunctiva. Her blood pressure was 100/60 mmHg, heart rate of 114 beats per minute, body mass index of 23 kg/m2, and SPO2 of 92%. There was decreased air entry and fine crepitations on the left side in the lower lung zone. Abdominal examination was significant for only mild distention. She had tenderness and swelling in her metacarpophalangeal and interphalangeal joints, and there was mild tenderness in the knee joints. A comprehensive examination of the other systems showed no abnormalities.

### Diagnostic approach

The patient's diagnosis presented significant challenges, especially since electronic medical records were not widely available at the hospital where she received treatment. Examining her case, starting from her last postpartum period and tracing the sequential events leading up to her unfortunate death, facilitates a clearer understanding of her condition.

The patient’s last postpartum period after cesarian delivery was followed by severe abdominal pain, nausea, and vomiting that improved with conservative treatment. However these symptoms recurred after 5 days, and an ultrasound was performed that suggested a liver abscess. However, when an abdominal computed tomography (CT) scan was performed, a diagnosis of cholecystitis was made, and she received conservative treatment in the surgical unit and was discharged within a day when her symptoms improved. However, after 1 day of being discharged, she was readmitted again to a neurology ward due to abnormal body movement, headache, altered mental status, and a high fever. She was diagnosed with posterior reversible encephalopathy syndrome by the neurologist after brain magnetic resonance imaging revealed white matter vasogenic edema.

On 24th of November 2023, on her second day in the neurology ward, due to complaints of palpitations, sweating, and abdominal pain, an electrocardiogram (ECG) was done, and it showed sinus tachycardia and ST segment depression. The electrolyte levels throughout the patient's clinical course are summarized in (Table 1). High-sensitivity cardiac troponin T was ordered, and it showed a 139 pg/ml level (normal reference range <14 pg/ml). It was ordered again after 2 hours, and it was stable at 138 pg/ml. Echocardiography showed all normal parameters. To further investigate the patient's condition, CT pulmonary angiography was done, and it showed no filling defect, but it was significant for bilateral lower lobe basal atelectasis, interstitial thickening, and subpleural fibrotic bands, in addition to right side mild pleural effusion. There was no abnormality on CT coronary angiography. Furthermore, magnetic resonance retrograde pancreatography (MRCP) revealed a 9 cm distended gallbladder, mild wall thickening, and regional effusion. A small 6 mm filling defect was seen at the neck, assumed to be due to a stone causing acute cholecystitis. Abdominal and pelvic CT showed a 5 × 1.5 cm collection just under the site of the cesarian section incision with multiple air bubbles inside. These findings suggested that an infected collection could be due to a foreign body from a surgical operation. It was associated with diffuse small intestinal dilation, possibly due to diffuse ileus. The gallbladder was distended, and a retroperitoneal inflammatory process was also noted with no specific lesion. The fluid collection was aspirated and sent for antimicrobial sensitivity testing, which revealed the growth of staphylococcus hemolyticus.

**Table 1:** The potassium, sodium, and chloride concentrations over the patient's clinical course.

Date	Potassium/normal range (3.5-5 mEq/L)	Sodium/normal range (135-147 mEq/L)	Chloride/normal range (96-106 mEq/L)
November 20, 2023	3.18	139.2	99
November 22, 2023	2.69	137.7	94.7
November 25, 2023	2.69	133.2	91.5
November 26, 2023	3.34	151.9	93.4
November 27, 2023	3.24	134.3	95.8
November 29, 2023	2.53	138	107.4
November 30, 2023	2.77	129	95.4
December 2, 2023	3.61	130.6	100.2
December 4, 2023	3.67	130.4	95.6
December 6, 2023	3.3	138.8	102
December 8, 2023	3.59	143.5	103.7
December 21, 2023	4.08	134	96.7
December 31, 2023	2.47	140.1	98.7
January 3, 2024	3.51	145.1	88.5
January 4, 2024	3.39	134	95.7
January 5, 2024	2.98	129.5	88.8

On the 29th of November, 2023, Abdominal ultrasound showed a normal intrahepatic biliary tree. The common bile duct wall was collapsed and edematous, suggesting cholangitis; the gallbladder contained sludge and a 5mm edematous wall. In addition, it showed a 4 × 1.5 cm thick wall collection seen anterior to the uterus with central gas shadow. A striking finding was a 4 × 2.5 cm partly cystic lesion at the region of adrenals suggestive of adrenal hemorrhage. A repeat ultrasound after 6 days revealed a 6 × 2 cm fluid collection at the region of the right adrenal gland with septa at midportion, suggestive of an adrenal cyst or hemorrhage. No gallbladder edema, hepatobiliary abnormality, or fluid collection was to be seen near the uterus now. In order to further investigate the state of the adrenals, serum levels of Cortisol and Adrenocorticotrophic hormone (ACTH) were measured, and they were 188.2 µg/L (normal reference range: 100-200 µg/L) and 20.58 pg/ml (normal reference range: 7.2-63.3 pg/ml), respectively. On the 7th of December 2023, the blood film showed normochromic normocytic red blood cells with mild anisocytosis and increased rouleaux formation. Abdominal ultrasound this time detected no adrenal hemorrhage or abnormality. Echocardiography showed tachycardia, fair left ventricular systolic function, mild left atrial dilatation, and mild mitral regurgitation. D-dimer of 1880 ng/ml (normal reference range <500 ng/ml), and high sensitivity cardiac troponin T of 528 pg/dl. The rise in troponin levels from the previous time and the ECG findings with the exclusion of ischemic heart conditions were suggestive of myocarditis.

**Fig. 1:**
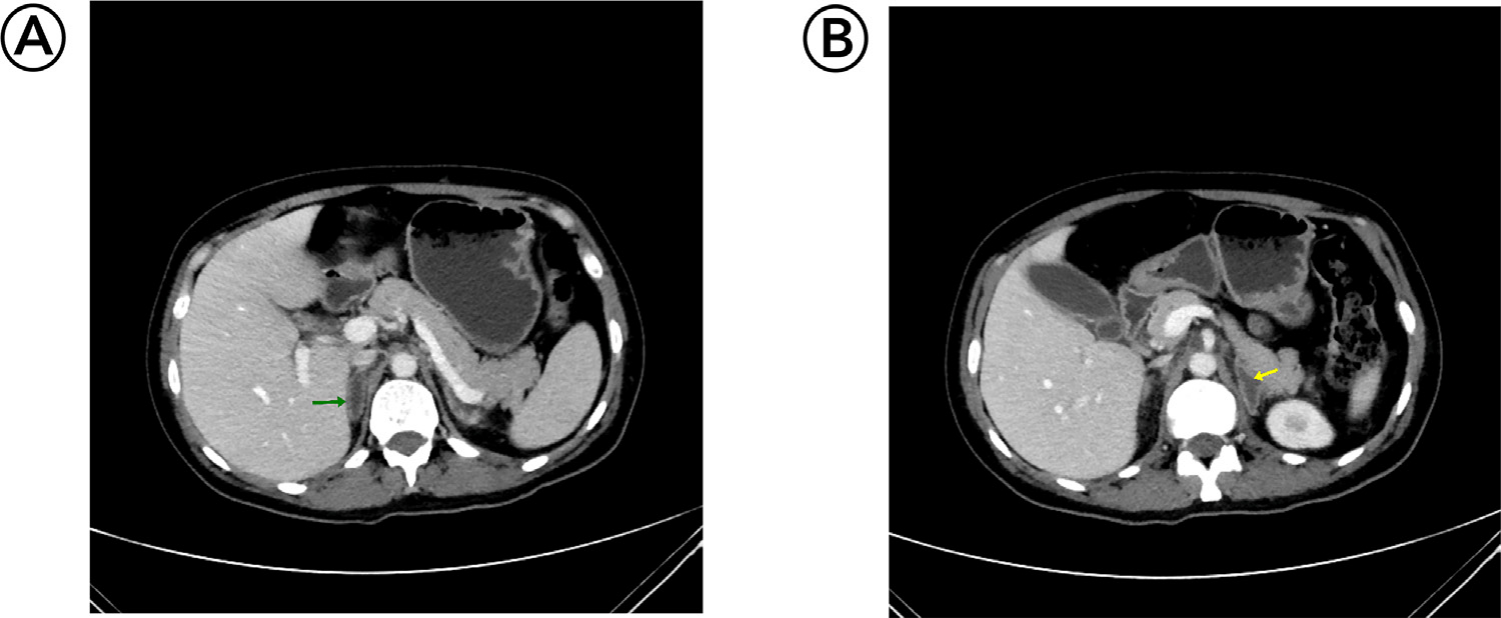
Axial sections of contrast-enhanced CT abdomen show central fluid density and peripheral enhancement of the right adrenal “green arrow” and left adrenal “yellow arrow,” indicating adrenal necrosis or hemorrhage.

On the 16th of December 2023, she was admitted again due to a fever, and the laboratory results were significant for low hemoglobin (HGB), hematocrit (HCT), and platelet count (9.4 g/dl) (normal reference range: 12.1-15.1 g/dl), (28.5%) (normal reference range: 36.0%-44.0%), and (120 × 10^9^/L) (normal reference range: 150 × 10^9^/L-450 × 10^9^/L), respectively with high C-reactive protein (CRP) (102.1 mg/L) (normal reference range <10 mg/L), and erythrocyte sedimentation rate (ESR) (91 mm/hr) (normal reference range <20 mm/hr). White blood cell count was normal. A general urine examination revealed turbid urine with +++ RBC, and a few pus cells and bacteria were seen, but the other parameters were normal. On the 20th of December 2023, a blood film showed normocytic normochromic anemia with prominent rouleaux formation. Blood culture results showed no growth, but urine culture showed streptococcus agalactia growth. Abdominal CT scan showed bilateral heterogeneous enlargement of both adrenal glands and their substance replaced by fluid (Fig. 1). ACTH stimulation test was done, and the results were as follows: Basal cortisol was (146 µg/L), basal ACTH (446 pg/ml), cortisol after 30 min (138 µg/L), cortisol after 60 min (147 µg/L). The level of Dehydroepiandrosterone sulfate (DHEA-S) was measured, and it was (1.47 mcg/dl) (normal reference range: 35-430 mcg/dl), which confirmed the adrenal insufficiency in this patient.

On the 25th of December 2023, blood investigation results were as follows: HGB (8.5 g/dl), HCT (25.9%), platelet (72 × 10^9^/L), CRP (35 mg/L), ESR (120 mm/hr). PT, PTT, and INR were all normal. Anticardiolipin IgM and IgG antibodies were normal. After 1 day, further results came back as follows: positive antinuclear antibodies (ANA) and Anti-cyclic citrullinated peptides (anti-CCP) antibodies of (1.7 IU/ml) (normal reference range ≤ 7 mg/L), and (102.9 AU/ml) (normal reference range < 20 AU/ml) respectively. Lupus anticoagulant sensitive PTT (66.5 sec) (normal reference range: 30.3-34.9 sec), Diluted Russell Viper Venom Time (dRVVT) screen (57.3 sec) (normal reference range: 30-42 sec), and dRVVT screen ratio (1.69) (normal reference range <1.20) were all positive. In addition to low C4 (16 IU/L) but normal C3 and creatine kinase-myoglobin binding (CK-MB) levels. The general urine examination was repeated, and it was insignificant with normal renal function tests. The diagnosis of systemic lupus erythematosus was made, but the patient did not complete the diagnostic criteria of APS.

On the 1st of January 2024, the patient experienced a sudden and rapid deterioration in her health status. The manifested symptoms were palpitations, dyspnea, hypoxia, sweating, and diffuse chest crackles. ECG showed nonspecific ST segment and T wave changes with normal QT interval. Echocardiography revealed reduced left ventricular systolic function, ejection fraction of 35% (normal reference range: 55%-70%), septal wall dyskinesia with global wall hypokinesia, dilated right atrium and ventricle with impaired right ventricular systolic function, and moderate tricuspid regurgitation. It further showed that her systolic pulmonary artery pressure was 55 mmHg. Pulmonary arteries on CT angiography showed a filling defect involving the main pulmonary arteries, branches of both main pulmonary arteries, lower interlobular, and segmental branches, still with contrast flow distal to the filling defects suggestive of acute pulmonary thromboembolism. After 1 day, repeat CT pulmonary angiography revealed right and left pulmonary arteries, as well as most of the distal pulmonary artery trees, with linear filing defects, mainly on the right side, suggestive of a severe form of pulmonary embolism. Diffuse ground glass changes and early signs of consolidation affecting both lung parenchyma in addition to a right-side pleural effusion were seen as suggestive of pulmonary infarction with possible pneumonic changes. The laboratory investigations were as follows: HGB (6.2 g/dl), HCT (19.8%), platelet (50 × 10^9^/L), WBC (22.9 × 10^9^/L) (normal reference range: 4.5-11.0 × 10^9^/L), Alkaline phosphatase (ALP) (138 U/L) (normal reference range: 44-147 U/L), Alanine aminotransferase (ALT) (68 U/L) (normal reference range: 7.0-45.0 U/L), Aspartate aminotransferase (AST) (78 U/L) (normal reference range: 8.0-33.0 U/L), CRP (455 mg/L), Thyroid function tests normal except for low T3 (0.64 nmol/L) (normal reference range: 1.2-2.7 nmol/L).

### Therapeutic intervention

Throughout the patient's long and complicated course of events, the patient received various treatments from several physicians depending on the time and cause of admission. The medications that she received in the surgical ward from 21st to 26th November 2023 included esomeprazole, paracetamol, ceftriaxone, metronidazole, and metoclopramide, in addition to aspirin, atorvastatin, clopidogrel, and heparin after the cardiology consultation on the 24th of November. In the neurology department, she received lisinopril, amlodipine, esomeprazole, ceftriaxone, enoxaparin, metoprolol, metoclopramide, paracetamol, diazepam on need, lactulose and bisacodyl for constipation. When the results of her culture and sensitivity returned in the internal medicine ward, her medications included meropenem, enoxaparin, potassium chloride, 1500 cc normal saline, omeprazole, metronidazole, and pethidine 50 mg since she was not responding to tramadol. Subsequently, owing to the patient's financial constraints, the meropenem was replaced with vancomycin and moxifloxacin. When the patient was diagnosed with adrenal insufficiency, she was put on hydrocortisone 100 mg twice daily, heparin 1 cc twice daily, cefotaxime, paracetamol, omeprazole, diclofenac, 1000 cc ringer lactate. After 5 days, the heparin was stopped after consultation with a hematologist due to low platelet levels and suspected heparin-induced thrombocytopenia; the heparin was switched to apixaban 5 mg twice daily, and the patient also received plasma. In addition, methylprednisolone 500 mg, hydroxychloroquine 200 mg twice daily, naproxen, and omeprazole were prescribed. Two pints of blood and Rituximab 1g were given for the debatable catastrophic APS on the third of January.

### Follow-up and outcome

On the fifth of January, the patient's condition deteriorated rapidly, likely due to a combination of her adrenal insufficiency and concurrent myocarditis. Despite resuscitative efforts, she passed away.

## Discussion

Systemic lupus erythematosus (SLE) is a chronic autoimmune disorder presenting with a broad spectrum of symptoms, varying in severity from mild to severe. Multiple organ systems may be implicated in its pathogenesis [1]. It is often associated with APS, which affects around 20%-30% of SLE patients [5]. Even though the current patient was diagnosed with SLE per the 2019 American College of Rheumatology criteria, sensitivity of 96% and specificity of 93%, by fulfilling the entry criterion of having a positive ANA test in addition to arthralgia, fever, thrombocytopenia, seizure, pleural effusion, and a low C4 level [9]. But despite a thorough consideration of APS in the context of SLE in this patient, especially with a history of 2 miscarriages and the patient’s clinical course being complicated by pulmonary embolism and adrenal hemorrhage, which is amongst the clinal domains of the 2023 American College of Rheumatology criteria for APS, sensitivity of 84% and specificity of 99%, but the present patient failed to complete the criteria due to insufficient points for the laboratory domains, moreover, it did not meet the revised Sapporo criteria which has a higher sensitivity of 99% for APS [4,10].

A literature review summarizing several cases similar to the current patient's condition is presented in (Table 2) [5,[11], [12], [13], [14], [15], [16]]. The clinical manifestations of adrenal hemorrhage may display notable variation. Symptoms may involve nausea, vomiting, sudden abdominal pain, palpable loin mass or pain, high or low blood pressure, and altered mental status. Laboratory results may be within the normal ranges, or it might present with a notable reduction in hemoglobin levels or alteration in electrolyte levels due to adrenal insufficiency if extensive damage is done to the parenchyma. These symptoms are frequently misinterpreted as indicative of sepsis, especially in the context of a complicated and clinically unwell patient. In the majority of instances, adrenal hemorrhage occurs unilaterally, allowing the unaffected contralateral adrenal gland to maintain normal function. Even in cases of bilateral hemorrhage, a significant proportion of patients may not exhibit symptoms of adrenal insufficiency. The current case had significantly low hemoglobin levels that required blood transfusion along with unstable electrolyte levels throughout the hospital stay. Adding to the rarity of this case, she had bilateral adrenal hemorrhage on abdominal CT scan, which is the prevailing imaging modality typically acquired in instances of adrenal hemorrhage, with symptoms of adrenal insufficiency, which included darkened skin, fatigue, and hypotension. The primary adrenal insufficiency was diagnosed as such since the basal cortisol levels and the cortisol levels after the ACTH stimulation tests were indeterminate. The assay type becomes crucial in interpreting these values; however, when the assay type can’t be verified, then requesting a DHEA-S level becomes paramount in identifying the presence of adrenal insufficiency, which, in this case, the primary adrenal insufficiency was diagnosed by the low DHEA-S level. [6].

**Table 2:** Characteristics of patients with adrenal hemorrhage and systemic lupus erythematosus.

References	Age (years)	Sex	Underlying Connective tissue disease	Symptoms	Serum cortisol (50-200 µg/L)	ACTH level (<46 pg/ml)	Other Laboratory results	Radiological finding (CT or MRI)	Medication received	Outcome	Significant patient details
Zhang et al. [11]	35	F	SLE	Arthritis, fever, facial rash, photosensitivity, Raynaud’s phenomenon, hyperpigmentation	31.5	14,800	LA, anti-dsDNA, anti-RNP, ACL, ANA, and anti-SSA were all positive. Hypocomplementemia, Thrombocytopenia, proteinuria	Adrenal hemorrhage	Unspecified	Alive	-
Abdulla et al. [12]	24	F	SLE	Intermittent headache; lower abdominal pain; vomiting; pale, icterus and mild splenomegaly	1.8	-	Anti-dsDNA and ANA were positive HGB: 3.1 g/dl, leucocyte count 4200/ml, platelet count 0.76 × 109 /L hypocomplementemia,	Bilateral adrenal hemorrhage	Steroids	Alive	-
Bouki et al. [13]	30	F	SLE + APS	Diffuse abdominal pain, general weakness, arthralgias, hyperpigmentation, nausea, vomiting, anorexia, and unintentional weight loss of 48 kg over a 5-month period. She complained of watery diarrhea for 4 weeks and secondary amenorrhea for the last 3 months	1	-	Anti-ds-DNA, Anti-β2-GP1 IgM, aCL IgM, and LA were all positive DHEAS: 5.7 mcg/dl. Troponin I: 1916 pg/ml Sodium 125 mEq/L, Potassium 6.1 mEq/L Creatinine 5.84 HGB: 9.8 g/dl, aPTT 40.5, PT 20.5, INR 1.75,	Bilateral adrenal hemorrhage (the left measuring 2.55 cm and the right 1.75 cm	Hydrocortisone, norepinephrine, antibiotics, blood transfusion, antiarrhythmics, LMWH,	Alive	2 years prior SLE initially manifested by myocarditis-induced cardiopulmonary arrest and secondary APS with recurrent episodes of unprovoked DVT and sub-segmental pulmonary embolism
Xu et al. [14]	45	M	SLE + APS	Fatigue, loss of appetite, nausea and vomiting for 2 months, hypotensive and hyponatremic	8.3	558	LA, anti-dsDNA, aCL IgM, and ANA were all positive Sodium 117.9 mEq/L, aPTT 95 sec, leukocyte count 2.36 × 109 /L, platelet of 81 × 109 /L, hypocomplementemia	Bilateral adrenal hemorrhage	Methylprednisolone, heparin, cyclophosphamide, hydroxychloroquine, warfarin, oxcarbazepine, entecavir	Alive	Positive history of epilepsy
Diana et al. [15]	62	M	SLE + APS	Weakness, and diffuse abdominal pain for 1 week. 5kg weight loss in 1 month. Hyperpigmentation, Altered mental status and hypotensive	<10	>1250	LA, anticardiolipin antibodies, and anti-β2 glycoprotein were all positive Sodium: 122 mEq/L Potassium; 6.3 mEq/L, Platelet; 130 × 109 /L	Bilateral adrenal hemorrhage in subacute phase	Hydrocortisone, fludrocortisone, azathioprine, hydroxychloroquine, levothyroxine,	Alive	Subclinical hypothyroidism,
Bansal et al. [16]	50	M	APS	Acute left lower extremity pain and edema (DVT), fever, pleuritic chest pain and dyspnea (PE), later developed bilateral flank pain and hypotension	3.3	319	ANA, anti-dsDNA aCL, and anti-SSA were all positive	Bilateral adrenal hematoma	Hydrocortisone, Rivaroxaban, heparin, and warfarin	Alive	-
Jiang et al. [5]	32	F	SLE + APS	Hyperpigmentation, limb weakness, abdominal pain, low appetite.	3.1	>1250	LA and anti-dsDNA, anti-histone, aCL, and anti-β2 glycoprotein I antibodies were all positive	Bilateral adrenal hemorrhage	Hydrocortisone, hydroxychloroquine, aspirin,	Alive	-

Abbreviations: ACTH, adrenocorticotropic hormone; aCL, anticardiolipin; ANA, antinuclear antibody; APS, antiphospholipid syndrome; aPTT, activated partial thromboplastin time; CT, computed tomography; DHEAS, dehydroepiandrosterone sulfate; DVT, deep vein thrombosis; HGB, hemoglobin; INR, international normalized ratio; LA, lupus anticoagulant; LMWH, low molecular weight heparin; MRI, magnetic resonance imaging; PE, pulmonary embolism; PT, prothrombin time; SLE, systemic lupus erythematosus; SLE + APS, systemic lupus erythematosus + antiphospholipid syndrome; Anti-dsDNA, anti-double stranded DNA; Anti-RNP, anti-ribonucleoprotein; anti-SSA, Anti-Sjögren's Syndrome-related Antigen A; Anti-β2-GP1, anti-β2 Glycoprotein I.

In a comprehensive review encompassing 85 patients presenting with adrenal involvement secondary to APS, a significant proportion (70%) exhibited primary APS, with 16% demonstrating SLE accompanied by secondary APS [17]. As such, in cases of SLE, the occurrence of bilateral adrenal hemorrhage may indicate the presence of coexisting APS. For instance, Jiang et al. presented a case involving a 32-year-old female with a background of SLE who exhibited symptoms of abdominal discomfort, limb weakness, and reduced appetite. Following a comprehensive evaluation that detected hyponatremia, decreased plasma cortisol levels, and elevated adrenocorticotropic hormone levels, the patient received a diagnosis of adrenal hemorrhage associated with secondary APS The patient was treated with hydrocortisone and warfarin. [5]. Bansal et al. documented a case of adrenal insufficiency caused by bilateral adrenal hemorrhage in a 50-year-old male diagnosed with primary APS. The patient initially presented with deep venous thrombosis and pulmonary embolism, later experiencing hypotension and bilateral flank pain attributed to the bilateral adrenal hemorrhage [16]. Although several case reports, as mentioned, address bilateral adrenal hemorrhage in patients with APS with or without SLE, there are very few documented cases of adrenal hemorrhage in solely SLE patients in the literature. Zhang et al., in a case series of 6 patients, reported one individual with confirmed SLE and bilateral adrenal hemorrhage on imaging with darkening of palmar creases, lips, and mucous membranes [11]. Additionally, Abdulla et al. described a case of a 24-year-old female with SLE who presented with rare hemorrhagic complications, including bilateral adrenal, subdural, and soft tissue hemorrhages. Despite the presence of bilateral adrenal hemorrhage, APS antibodies were negative. The patient was treated with high-dose steroids and showed improvement [12]. However, the present patient’s condition deteriorated despite multiple efforts of treatment through the patient's disease course by methylprednisone, hydrocortisone, hydroxychloroquine, and even rituximab with no success. The rapid deterioration of the patient raised concerns regarding catastrophic APS, which was considered in the clinical evaluation of the case. However, it did not fulfill the definite nor the probable diagnostic criteria for catastrophic APS [18].

The pathophysiology of adrenal hemorrhage and the possible hypotheses of its occurrence require an understanding of its anatomy. Within the adrenal glands, drainage occurs through a solitary central vein, notable for its dense longitudinal muscle bundles, which contribute to heightened resistance against blood flow. Elevations in adrenal venous pressure or arterial perfusion pressure can lead to hemorrhage within the gland. During episodes of hypotension and reduced arterial perfusion, capillaries located at the corticomedullary junction become vulnerable to ischemic necrosis. Upon restoration of normal arterial perfusion, reperfusion injury may occur, leading to hemorrhage. One hypothesis is that in times of physiologic stress, elevated ACTH levels trigger the secretion of catecholamines, enhancing blood circulation to the adrenal gland. Enhanced arterial flow, along with the vasoconstriction induced by catecholamines in the adrenal vein, culminates in heightened pressure within the adrenal gland, potentially leading to hemorrhage. Even though the current patient had evidence of bilateral adrenal hemorrhage prior to the ACTH stimulation test, there is still a possibility that the ACTH stimulation test could have theoretically led to more bleeding based on the aforementioned hypothesis. A more specific hypothesis for the occurrence of adrenal hemorrhage in patients of APS is that due to the hypercoagulable state, a thrombus might form in the adrenal veins and lead to increased backward pressure into the parenchyma and cause hemorrhagic infarction and bleeding in the adrenal glands. The use of anticoagulants such as heparin can be a cause of adrenal hemorrhage either due to direct bleeding complications or due to heparin-induced thrombocytopenia leading to thrombosis in the adrenal veins. However, this is unlikely in this case as she had normal platelet levels when the adrenal hemorrhage was detected, but later, she developed a low platelet count, and heparin-induced thrombocytopenia was suspected, but due to the financial crisis in the country where the patient lived; confirmatory serotonin release assay and other investigations were not available [6].

Another complication that this patient suffered from was myocarditis which is an infrequent occurrence in SLE patients. However, the deterioration of the current patient was in line with the detection of echocardiographic findings of ejection fraction of 35% and septal wall dyskinesia with global wall hypokinesia, in addition to high troponin titers and lack of evidence for ischemic heart conditions on angiography. Even though peripartum cardiomyopathy cannot be excluded definitively, lupus myocarditis seems more probable, considering the patient's SLE diagnosis. This clinical presentation aligns with an exacerbation of SLE complicated by lupus myocarditis, similar to the findings in the case of Batt et al., who was an SLE patient with bilateral adrenal infarction that was later complicated by lupus myocarditis and had high troponin titers with hypokinesia on echocardiography. However, their case improved after she commenced treatment with hydroxychloroquine, intravenous methylprednisolone, and intravenous immunoglobulin. In contrast to the current case, the patient did not experience improvement with the administered medications and later died [19].

In conclusion, patients with an SLE flare can develop bilateral adrenal hemorrhage and cause adrenal insufficiency even in the absence of definitive APS. The development of lupus myocarditis in such patients may result in worsened outcomes and potentially death.

## Availability of data and material

All data and materials are kept by the first and corresponding authors.

## Patient consent

Consent has been taken from the patient.
